# Emerging treatment strategies for glioblastoma multiforme

**DOI:** 10.15252/emmm.201302627

**Published:** 2014-10-13

**Authors:** Steven K Carlsson, Shaun P Brothers, Claes Wahlestedt

**Affiliations:** 1 Department of Psychiatry and Behavioral Sciences, Center for Therapeutic Innovation, University of Miami Miller School of MedicineMiami, FL, USA

**Keywords:** biomarkers, brain imaging, cancer stem cells, epigenetics, glioblastoma multiforme (GBM)

## Abstract

Glioblastoma multiforme (GBM) is the deadliest form of brain tumor with a more than 90% 5-year mortality. GBM has a paltry median survival of 12.6 months attributed to the unique treatment limitations such as the high average age of onset, tumor location, and poor current understandings of the tumor pathophysiology. The resection techniques, chemotherapic strategies, and radiation therapy currently used to treat GBM have slowly evolved, but the improvements have not translated to marked increases in patient survival. Here, we will discuss the recent progress in our understanding of GBM pathophysiology, and the diagnostic techniques and treatment options. The discussion will include biomarkers, tumor imaging, novel therapies such as monoclonal antibodies and small-molecule inhibitors, and the heterogeneity resulting from the GBM cancer stem cell population.

## Introduction

Gliomas are the most commonly occurring form of brain tumor. Glioblastoma multiforme (GBM) is the most malignant form of glioma causing 3–4% of all cancer-related deaths (Louis *et al*, 2007). The World Health Organization defines GBM as a grade IV cancer characterized as malignant, mitotically active, and predisposed to necrosis. GBM has a very poor prognosis with a 5-year survival rate of 4–5%, which perhaps is an overestimation (McLendon & Halperin, 2003). GBM has a paltry median survival of 12.6 months attributed to unique treatment limitations such as a high average age of onset, tumor location, and poor current understandings of the tumor pathophysiology (Louis *et al*, 2007). Current standard of care for GBM includes tumor resection with concurrent radiotherapy and chemotherapy. However, no marked improvements have been achieved that increase survival rates close to other glioma subtypes (Stewart, 2002). The development of proteomic, genetic, and epigenetic tools highlighted here may hold the potential to improve survival rates for GBM patients.

## The diagnosis of GBM

### Medical imaging

For the last 20 years, magnetic resonance imaging (MRI) has been the standard in brain tumor imaging to define lesion boundaries including size, shape, and location of the tumors. There are, however, a number of emerging MRI developments with the potential to provide more detail about the structural changes that differentiate the higher-grade glioma subtypes. For example, advanced diffusion-weighted imaging can differentiate soft tissue populations based on cellular density, thus discriminating GBM from malignant lymphoma (Yamasaki *et al*, 2005). Such techniques provide useful information beyond the initial tumor diagnosis. Perfusion weight imaging can be used to monitor the clinical effectiveness of anti-angiogenic drugs like bevacizumab through the use of post-treatment parametric response map comparisons (Aquino *et al*, 2014). Relative cerebral blood volume (rCBV), a measure of microvascular density, is decreased in patients that responded to the anti-angiogenic treatments. Elevated rCBV values also correlate with *EGFR* amplification which may have prognostic and treatment monitoring applications in the future (Gupta *et al*, 2014). Despite the well-characterized diagnostic and treatment applications of MRI, tumor assessment is still largely confined to the evaluation of changes in brain anatomy and structure, which does not profile real-time tumor dynamics.

Methods of identifying solid tumors based on alterations of metabolism are now being actively developed. As first observed by Otto Warburg in 1927, cancer cells switch glucose metabolism favoring glycolysis followed by lactic acid production over oxidative phosphorylation, even in the presence of sufficient oxygen (Warburg *et al*, 1927). Although this aerobic glycolysis is less efficient than oxidative phosphorylation, higher biomass incorporation through glycolysis is advantageous to proliferating cancer cells by providing the necessary organic substrates for nucleic acid and lipid synthesis. About 90% of all glucose consumed in glioblastoma cells is converted to lactate or alanine, which may be useful to differentiate GBM tumors from surrounding tissue (DeBerardinis *et al*, 2007). In this respect, the emergence of positron–emission tomography (PET) has been critical. High-grade glioma can be differentiated from CNS lymphoma using the tumor's ability to avidly uptake the glucose analog deoxy-2-(^18^F)fluoro-D-glucose (FDG) (Kosaka *et al*, 2008). Increased FDG uptake negatively correlates with patient survival in glioma patients (Pardo *et al*, 2004). In GBM and in general brain tumors, however, the poor signal-to-noise ratio due to the elevated glucose uptake in the brain compared to other tissues somewhat limits the usefulness of FDG. In fact, radiolabeled amino acids such as C-methionine (C-MET) are preferred over FDG due to their lower uptake in normal brain cells (Stolc *et al*, 2011). C-MET is actively transported into proliferating cells through the carrier-mediated transport activity of LAT1, which is upregulated in malignant glioma (Kobayashi *et al*, 2008). C-MET and not FDG was found to be a significant prognostic factor for GBM based on uptake in proliferative cells (Kim *et al*, 2005). As far back as 1986, pathological histology has defined GBM boundaries beyond those determined through MRI (Lunsford *et al*, 1986). While C-MET is likely a better imaging technique than FDG, the use of C-MET may be advantageous over conventional MRI as well. MET PET imaging substantially enlarges the three-dimensional volume of an active tumor in comparison with gadolinium–DTPA-enhanced MRI (Galldiks *et al*, 2010). In conclusion, while MRI remains the standard for GBM brain imaging, advances in technology and wider availability support the use of PET as a complementary technology.

### Biomarkers

While GBM tumors may present similar or overlapping phenotypes, differences in tumor progression and molecular mechanisms require diagnosing beyond histological profiling. Histological profiling per se presents a challenge as the required resection or biopsy can be problematic given the location and sensitive nature of brain tissue. Clearly, non-invasive diagnostic measures able to better identify and differentiate GBM tumors would expedite non-resection therapeutic methods ultimately improving patient survival.

Biomarkers are potentially a non-invasive and universal diagnostic tool that focuses on molecular markers over the phenotypic differences described through biopsy. GBM microvesicles may prove to be useful in this respect. Microvesicles are plasma membrane-derived membrane-enclosed particles that are released from cells through membrane fission and can carry mRNA, miRNA, and proteins from the parent cell (Cocucci *et al*, 2009). When these microvesicles are GBM derived, the tumor-specific contents can adjust the nearby microenvironment to be more hospitable to tumor growth (Skog *et al*, 2008). One example is the GBM driver mutant form of the EGF receptor EGFRvIII, which can induce neighboring cells to transform into GBM-like phenotypes. Microvesicles carrying EGFRvIII can transfer the oncogenic receptor to induce EGFRvIII activity in the receiving cell (Al-Nedawi *et al*, 2008). Patient serum may provide prognostic information as it has also been used to detect a small non-coding RNA, RNU6-1, which is an independent predictor of GBM (Manterola *et al*, 2014). Clearly, the serum composition of GBM patients should be further studied as it may non-invasively provide highly valuable prognostic information for treating GBM beyond the current paradigms.

Microfluidic chips can be used to detect microvesicles in the bloodstream and have been shown to detect a significant dose-dependent post-temozolomide (TMZ) treatment decline in total microvesicle populations (Shao *et al*, 2012). In addition, microvesicles can accurately model the profile of the tumor cell including changes in IDH-1, EGFR, and EGFRvIII. The information provided by microvesicle detection may provide a quick and non-invasive biomarker of GBM status using patient blood samples. Microvesicles and mRNA are not the only potential biomarkers; however, a number of peptides have been found to change in CSF samples between normal and GBM patients (Schuhmann *et al*, 2010). Elevations in albumin, osteopontin, and others, although not GBM specific, might suggest that peptide levels in the CSF reflect changes in the nervous system environment that could be used to determine the status of a GBM tumor. Routine blood sampling after GBM resection may allow early detection of recurrence, thus reducing the time from tumor regrowth to second-line treatment.

## GBM molecular pathophysiology

### Epidermal growth factor receptor

A common driver of GBM progression is epidermal growth factor receptor (*EGFR*) amplification, found in nearly 40% of all GBM cases (Hatanpaa *et al*, 2010). *EGFR* phenotypic changes in GBM can occur by overexpression, amplification, and mutation. Amplification of *EGFR* can occur by reverse transcription from RNA or insertion, for example. Essentially, all cases of *EGFR* amplification in GBM are accompanied by *EGFR* overexpression, contrasted to the 97.7% of non-amplified *EGFR* GBMs that instead have no EGFR overexpression (Shinojima *et al*, 2003).

Amplification of *EGFR* is associated with the presence of EGFR protein variants. In 68% of EGFR mutants, there is a deletion in the N-terminal ligand-binding region between amino acids 6 and 273 termed EGFRvIII. Deletion in the ligand-binding domains of EGFR can lead to ligand-independent activation of EGFR (Yamazaki *et al*, 1990). Due to the specific nature of these exon 2–7 deletions in EGFRvIII, common tyrosine kinase inhibitors such as gefitinib have limited therapeutic use (Schulte *et al*, 2013). Therefore, approaches to address the lack of extracellular receptor are being pursued. EGFRvIII is implicated in the PKA-dependent phosphorylation of DOCK180, a guanine exchange factor for Rac1. Overexpression of mutant DOCK180 lacking the phosphorylation site at S1280 in an EGFRvIII-containing cell line inhibited receptor-stimulated proliferation and survival (Feng *et al*, 2014). This EGFRvIII/PKA/DOCK180 interaction may offer a unique therapeutic target if EGFRvIII-specific PKA phosphorylation can be inhibited. However, EGFRvIII is not prognostic of overall median survival except in cases of survivors of ≥ 1 year which may limit the therapeutic value of this target (Heimberger *et al*, 2005).

### p53 and PTEN

p53 is a well-known tumor suppressor protein that plays a fundamental role in the formation of high-grade tumors (England *et al*, 2013). p53 initiates DNA repair, or apoptosis if DNA damage is irreparable. There is a strong correlation between the presence of mutant p53 and the transition from low-grade astrocytoma to the high-grade glioblastoma (Sidransky *et al*, 1992). p53 mutant cells are more likely to expand to high-grade glioma as these cells outgrow and overtake the non-p53 mutant cell population (Sidransky *et al*, 1992). There is evidence that nuclear localization is correlated with long-term survival rates as nuclear p53 is responsible for apoptotic induction limiting tumor cell expansion. Long-term survivors (> 3 years) have tumors with high levels of nuclear p53 compared to short-term survivors. This is not caused by differences in the mutation rate (Burton *et al*, 2002). Recent gene therapy experiments with nanoparticle delivery of the p53 gene targeting glioblastoma and cancer stem cells showed induction of apoptosis after standard chemotherapy (Kim *et al*, 2014b) and improved survival in a mouse model. To date, this has not been tested in a clinical trial.

Multiple concurrent tumor suppressor mutations are common in GBM progression. One study found that primary tumors expressing mutant p53 had concomitant PTEN mutations or deletions in 6 out of the 10 samples (Zheng *et al*, 2008). PTEN is a phosphatase tumor suppressor critical in cellular homeostasis that is mutated in between 5 and 40% of GBMs and can be a prognostic indicator in patients > 45 years old (Srividya *et al*, 2011). Under normal conditions, PTEN facilitates homeostasis by preventing cell cycle entry, thus maintaining the neural stem cell population. Unsurprisingly, PTEN null mutants are more sensitive to growth factors and more prone to proliferation than wild-type neural stem cells (Groszer *et al*, 2006). Diagnostically, PTEN may turn out to be a valuable marker as PTEN levels are positively correlated with patient survival (Ermoian *et al*, 2002). The Cancer Genome Atlas (TCGA), a collaborative effort between the National Cancer Institute and the National Human Genome Institute, has elucidated PTEN mutations that may influence GBM development using genomic sequencing. The presence of PTEN non-sense mutations resulted in lower survival than wild-type in a murine xenograft model (Xu *et al*, 2014). Bryostatin, an inhibitor of PKC downstream of PTEN, suppressed tumor growth in the non-sense PTEN background suggesting that PTEN non-sense mutations can be indirectly targeted for treatment of GBM.

### Isocitrate dehydrogenase

**Figure 1:**
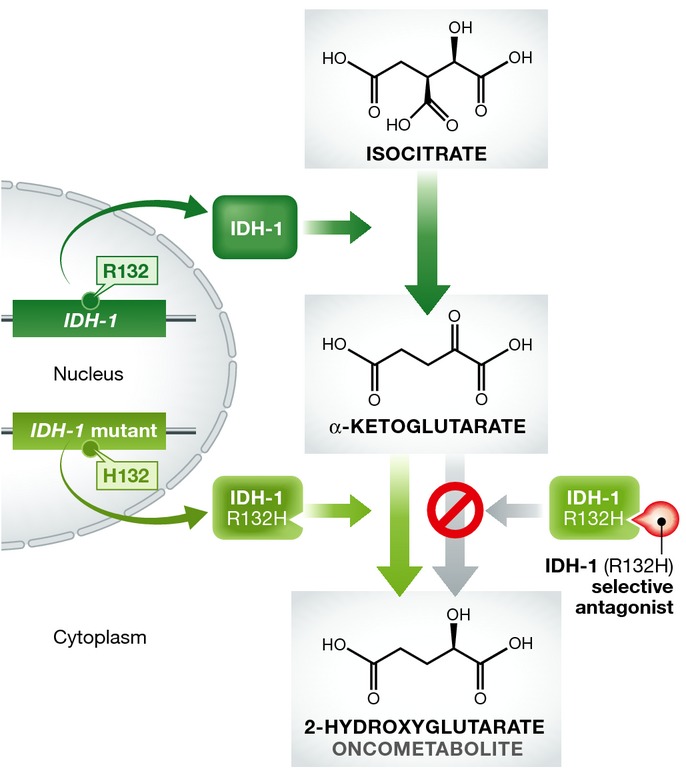
Inhibition of 2-hydroxyglutarate production in heterozygotic IDH-1 cells Wild-type IDH-1 converts isocitrate to alpha-ketoglutarate. Heterozygotic IDH-1 (R132H) mutant protein can convert alpha-ketoglutarate to 2-hydroxyglutarate, an onco-metabolite. Selective therapeutics against IDH-1 (R132H) prevent 2-hydroxyglutarate production while leaving normal IDH-1 enzymatic function intact.

The transition from low-grade gliomas to secondary GBM relies on the convergence of many pro-oncogenic events. In addition to the critical roles of PTEN and p53, isocitrate dehydrogenase (IDH-1) mutations are now considered to be a fundamental step in this transition. Although rare in primary GBM at a rate of 5%, this mutation is found in 83% of all secondary GBM cases (Kloosterhof *et al*, 2011). IDH-1 mutations are, in fact, now believed to be one the earliest events in the formation of low-grade gliomas preceding any mutation that may occur in the p53 gene (Watanabe *et al*, 2009). Mutations in IDH-1 exist as somatic point mutations and can simultaneously result in a reduction in enzyme efficiency or enzymatic gain of function depending on the substrate. The known point mutation resides in the active site, which prevents the enzyme from successfully converting isocitrate to alpha-ketoglutarate. More importantly, the arginine at codon 132 is replaced with a histidine in 90% of cases (Yan *et al*, 2009). The R132H mutation causes IDH-1 to gain the ability to convert alpha-ketoglutarate to 2-hydroxyglutarate (2HG), an onco-metabolite. Since IDH-1 mutations occur on one allele, this allows both normal and mutant IDH-1 to co-dimerize or act in *cis* to produce this onco-metabolite by converting isocitrate to 2HG in a two-step metabolism (Fig 1). Loss of wild-type IDH-1 when the R132H mutation is present on the other allele results in a 14-fold lower level of 2HG suggesting that both isoforms must be active for onco-metabolite production (Jin *et al*, 2013). 2HG may prove to be a suitable biomarker for the presence of IDH-1 mutations as 2HG levels can be detected using magnetic resonance (Kalinina *et al*, 2012). However, IDH-1 mutations have not been shown to effect median survival rate or progression-free survival in secondary GBM (Juratli *et al*, 2012). IDH-1 is now being targeted for therapeutic use for instance by Agios Pharmaceuticals, who are currently moving forward with the drug candidate AG-120 after their tool compound inhibitors were found to be successful in glioma xenografts (Rohle *et al*, 2013). AG-120 is currently undergoing a phase I trial (clinicaltrials.gov; NCT02073994).

### Genomics

Personalized medicine for GBM is evolving in part due to progress in genomic sequencing. TCGA has made the genomic databases of over 20 cancers public in an effort to enhance integrated analysis on a common data set. In 2008, TCGA detailed a multi-dimensional analysis of 216 cases of GBM that identified genetic changes that include inactivation of the Neurofibromin 1 gene, ERRB2 mutations, and MGMT methylation (Cancer Genome Atlas Research Network, 2008). TCGA GBM data set continues to provide new insights in the development of GBM. A recent evaluation of TCGA RNA-Seq data revealed a neurotrophic tyrosine kinase receptor type 1–neurofascin gene fusion in GBM. *In vitro*, this gene fusion showed increased proliferation of 3T3 cells, suggesting an oncogenic-like role (Kim *et al*, 2014a). Oncogenic gene fusion analysis is a growing field, made possible through the exploitation of large databases like TCGA.

However, TCGA GBM data set has use beyond targets of oncogenesis and has potential prognostic value. For example, although miRNA does not appear to add prognostic power to multidimensional genomic models in GBM (Zhao *et al*, 2014), the involvement of miRNAs in GBM is evident and has been greatly elucidated due to TCGA. Loss of *MIR-491* has been recently implicated in the proliferation and invasion of GBM. *MIR-491*, the gene encoding for miR-491-5p and miR-491-3p, is frequently deleted in GBM. This deletion is inversely correlated with the overexpression of EGFR, CDK6, and IGFBP2 (Li *et al*, 2014). In addition, the presence of functional *MIR-491* impairs the propagation of GBM cancer stem cells through the EGFR, CDK6, and IGFBP2 proliferative pathways. Reintroduction of *MIR-491* has promising therapeutic potential through the suppression of the proliferative pathways listed above. This depth of analysis would not have been possible without the volume of data available through TCGA.

miRNA pathways in GBM can influence the effectiveness of current treatments as with TMZ. For instance, miR-455-3p and miR-10a* confer cellular resistance to TMZ. Knockdown of either miR did not lead to cell death, but enhanced sensitivity to TMZ (Ujifuku *et al*, 2010). Other miRs such as miR-21 are differentially upregulated in GBM compared to lower-grade gliomas (Berthois *et al*, 2014). miR-21 is upregulated in response to TMZ and confers a certain degree of resistance to the drug, while the loss of miR-21 in TMZ-resistant cell lines resensitized them to the drug (Wong *et al*, 2012). Hence, while miR-21 inhibition or TMZ alone may be insufficient, their combination may significantly enhance cancer stem cell death (Zhang *et al*, 2012).

## Cancer stem cells

GBM, similarly to others, is a heterogeneous tumor comprised of many cell types. A 2004 study first identified a small CD133^+^ stem cell-like population in GBM responsible for the maintenance and proliferation of the tumor (Yuan *et al*, 2004). Transplantation of CD133-positive, but not CD133-negative, cells from patient biopsies in severely compromised immunodeficient mice produced a phenocopy of the patient's original tumor (Choy *et al*, 2012). Targeting CD133 is a potential therapeutic strategy to eliminate the cancer stem cell population. CD133 silencing in GBM-derived stem cells was shown to increase post-implantation survival in an *in vivo* mouse model (Brescia *et al*, 2013).

Normal neural stem cells rely on NOTCH signaling for cellular homeostasis (Alexson *et al*, 2006). Gamma-secretase-mediated inhibition of the NOTCH pathway depletes CD133 and blocks tumor growth *in vivo* and neurosphere formation in culture (Fan *et al*, 2010). CD133 cells can also be depleted by knockdown of BMI1, a transcription repressor that prevents stem cells from altering pluripotency, indicating the importance of CD133 for cancer stem cells.

There is a clear negative relationship between GBM progression and tumor location in the adult subventricular zone (SVZ). Forty-seven percent of patients with SVZ-located tumors experienced progression-free survival after 6 months after treatment compared to 69% of non-SVZ-contacted tumor patients (Jafri *et al*, 2013). Over 95% of GBMs expressed SSEA-1, a known stem cell marker in the SVZ (Son *et al*, 2009). SSEA-1^+^ cells are capable of differentiation and expansion in neurospheres similarly to CD133^+^ cells (Son *et al*, 2009). In addition to recurrence, SVZ-associated GBM cases also progressed more than their non-SVZ-located counterparts.

A new pathway has been discovered that links GBM stem cells to the development of GBM-specific endothelial cell-related pericytes (G-pericytes). Pericytes are core components of the neurovasculature critical to the maintenance of the blood–brain barrier (BBB) and microvasculature regulation. Pericyte-deficient mice have extensive endothelial cell hyperplasia in addition to morphological changes such as increased vessel diameter (Hellstrom *et al*, 2001). In addition, 14- to 16-month-old pericyte-deficient mice have a 20- to 25-fold plasma-derived immunoglobulin buildup in the cortex and hippocampus compared to age-matched controls suggesting blood–brain barrier breakdown (Bell *et al*, 2010). While these pericytes are beneficial to normal brain function, TGFβ induction of G-pericyte differentiation from the cancer stem cell lineage has been shown to preserve mutations present in the stem cell population, thus facilitating in tumor-specific growth (Cheng *et al*, 2013a). Pericyte signaling pathways are stimulated by hypoxic-specific exosomes released from GBM cells that trigger paracrine stimulation of pericytes by endothelial cells (Kucharzewska *et al*, 2013). This signaling pathway promotes pericytic release of regulatory factors such as VEGF-A to maintain proper vascular function. Thus, G-pericyte prevention or elimination may rescue ineffective angiogenic therapies for GBM that modulate VEGF-A.

## Metastasis

While lung or breast cancers often metastasize to the brain, GBM distal metastases are exceedingly rare and have only been reported in 0.44% of all cases (Robert & Wastie, 2008) The metastatic potential of GBM has nevertheless been recorded. For example, a case study found that two patients who had received organs from a single GBM donor eventually developed GBM metastases that led to their death. (Armanios *et al*, 2004). Most probably, the relative paucity of documented extracranial metastasis for GBM is in part due to the short lifespan of GBM patients. It is also possible that GBM cell escape is limited by the lack of lymphatic transport in the brain (Robert & Wastie, 2008). Indeed, the few reported metastasis cases were postulated to have been initiated by surgical resection causing BBB disruption (Schonsteiner *et al*, 2011). Clearly, GBM does have metastatic potential, similar to most other solid tumors, but this does not appear to be a significant factor in patient survival rates; as GBM-directed therapeutics improve patient life span, however, this may become an emerging issue.

## Current standard of care

### Resection

The current standard of care for GBM is maximally possible surgical resection of the tumor followed by combination radiotherapy and chemotherapy. Maximal resection for each tumor is case-specific based on tumor size, shape, and location of blood vessels, arteries, or sensitive brain regions. Surgical resection is generally classified as gross total resection (GTR), and subtotal resection (STR) when complete removal of the tumor is not met. Not surprisingly, one-year survival is significantly higher for patients with more than 90% tumor resection compared to those with less than 90% tumor resection (Orringer *et al*, 2012). It is thus essential that the surgeon's ability to resect the tumor as much as possible is improved to increase GBM patient survival. A current clinical trial is testing tumor-specific fluorescent staining to help surgeons differentiate between tumor and non-tumor cell tissue. 5-aminolevulinic acid (ALA) was the first attempt to show how this approach could aid in tumor resection (Stummer *et al*, 2000). ALA induces the accumulation of porphyrins specific to GBM that fluoresce under violet-blue light. The contrast in color between the porphyrin-containing tumor and adjacent normal tissue allows for more specific and thorough resection of tumor cells. ALA is likely to be very safe, with no associated deaths and only 1% of patients experiencing any neurological effects. Sixty-five percent of resections using ALA obtained GTR, while only 36% met GTR criteria using conventional methods (Stummer *et al*, 2006). Other fluorescent compounds are being tested to provide better resolution for optimal resection such as sodium fluorescein, which is both safe and feasible to use (Schebesch *et al*, 2013).

### Radiotherapy

When GTR is unfeasible, radiotherapy has been used in conjunction with surgery as early as 1979 when increasing levels of radiation applied to tumors were found to be dose-dependently correlated with median survival rates (Walker *et al*, 1979). Radiation therapy induces severe DNA damage causing the cells to undergo apoptosis due to double-strand breaks. Standard external beam radiation therapy includes six weeks of localized radiation therapy five times per week. Resistance to radiotherapy can be problematic in GBM as EGFRvIII confers cellular resistance to such treatment options by upregulating the DNA double-stranded break repair machinery (Mukherjee *et al*, 2009). Therefore, EGFRvIII inhibitors may increase overall tumor sensitivity to radiation therapy. While external beam radiation therapy (EBRT) is the standard of care, radio-surgical techniques have been developed to increase radiation therapy effectiveness in patients experiencing GBM recurrence. Gamma knife therapy delivers stereotactic high doses of radiation that confine treatment to the targeted GBM area. Gamma knife is considered as ineffective in the treatment of primary tumors due to the excessively large tumor volume. However, gamma knife monotherapy in a GBM mouse xenografts model increased survival (Skeie *et al*, 2013). This may be advantageous in future treatment regimens if routine biomarker analysis for recurrence is implemented. Quick identification of recurrence would allow for gamma knife treatment while the tumor remains volumetrically small.

### Chemotherapy

The current standard for chemotherapy for GBM is TMZ. First described in 2005, concurrent TMZ and radiotherapy increased median survival rates to 26.5% at 24 months, a vast improvement over the 10.4% with radiotherapy alone (Stupp *et al*, 2005). TMZ is a brain-penetrant alkylating agent that methylates purines (A or G) in DNA and induces apoptosis. TMZ is effective in a rechallenge setting where TMZ is reintroduced after a TMZ-free time period. TMZ rechallenge maintained similar progression-free rates seen in constant administration paradigms (Wick *et al*, 2009). However, as previously described, the genetic background of a given GBM greatly affects drug effectiveness. TMZ sensitivity was found to be correlated with the methylation state of *O*^6^-methylguanine-DNA methyltransferase (MGMT) promoter in cancer cells committed to differentiation and not in the stem-like progenitors. MGMT is a mediator of DNA mismatch repair that corrects TMZ-induced damage (Villalva *et al*, 2012). Lower cellular concentrations of MGMT due to gene silencing are correlated with higher sensitivity to TMZ and longer overall survival; this may be useful as a biomarker (Hegi *et al*, 2005; Esteller *et al*, 2000). Indeed, patients with MGMT gene silencing had survival rates of 21.5 versus 15.3 months. TMZ efficacy in stem cells may also be dependent upon the presence of MGMT. GBM cancer stem cells expressing MGMT did not respond as well as non-MGMT expressing cancer stem cells at the same dosage (Beier *et al*, 2008). The downside to TMZ use is the significant risk arising from TMZ-dependent DNA damage in healthy cells. This risk, combined with the possible inefficacy on GBM cells, strongly indicates that additional chemotherapy options are urgently required to improve both targeting of treatment to GBM cells and improving efficacy.

## Novel therapies

Although current therapy regimens have improved over the past 20 years, overall patient survival has not risen to the levels obtained for other solid tumors. New therapies with novel empirical designs are currently in clinical trials (Table1). All such therapies are designed for use in combination with the current standard of care as a means to improve treatment efficacy, and range from personalized medicine approaches targeting the tumor cells to the disruption of the tumor microenvironment.

**Table 1:** Potential GBM treatments currently in clinical trial.

Treatment	Intervention	Molecular target	Clinical phase
TRC105 + Bevacizumab (Avastin)	Antibody + Drug	Endoglin/VEGF	1
Amgen386	Antibody	Angiopoietin-1 and -2	1
AMG595	Antibody Drug Conjugate	EGFRvIII	1
PSMA ADC MMAE	Antibody Drug Conjugate	PSMA/Tubulin	2
Ketogenic Diet	Dietary Adjustment	N/A	1
Bevacizumab (Avastin) + TPI 287	Drug	VEGF/Tubulin	2
AR-67	Drug	Topoisomerase 1	2
PD 0332991 (Palbociclib)	Drug	Cyclin-dependent Kinase 4/6	2
Pazopanib (Votrient) + Topotecan (Hycamtin)	Drug	Tyrosine Kinase Receptors (multiple) + Topoisomerase 1	2
G-202	Drug	Sarcoplasmic/Endoplasmic Reticulum Calcium ATPase (SERCA) pump	2
Aldoxorubicin	Drug	DNA	2
Dovitinib (TKI258)	Drug	FGFR/VEGFR/PDGFR	1
AG-120	Drug	IDH-1 R132H	1
Axitinib (Inlyta) + Radiation Therapy	Drug + Radiation	Tyrosine Kinase Receptors (multiple)	2
NovoTTF-100A device + TMZ	Electrical device + Drug	N/A	3
DC-Vax L	Immunotherapy	N/A	3
HER2 Chimeric Antigen Receptor Expressing CMV-Specific Cytotoxic T Cells	Immunotherapy	N/A	1
Rindopepimut	Immunotherapy	EGFRvIII	3
Parvovirus H-1 (ParvOryx)	Virus	N/A	1
Live attenuated, oral (Sabin) serotype 1 poliovirus vaccine	Virus	N/A	1
DNX2401 and Temozolomide	Virus + TMZ	N/A	1

### Monoclonal antibodies

One of the leading new classes of therapeutic agents is based on the use of monoclonal antibodies that recognize cell surface receptors and ligands, to prevent receptor signaling through the disruption of receptor–ligand interactions and downstream receptor activation. FDA approved Avastin (bevacizumab), an antibody against vascular endothelial growth factor (VEGF), currently leads the way. GBM tumors, as they grow, secrete VEGF to promote neoangiogenesis. Systemic injection of Avastin aims to block the response to VEGF and thus prevent neovascularization of the tumor and consequently decrease its size (Ferrara *et al*, 2005). This treatment destabilizes the tumor microenvironment and does not target tumor-specific receptors or antigens, which is beneficial as the treatment is not restricted to a specific tumor type. There are, of course, side effects caused by a broad blockage of VEGF signaling, such as deep vein thrombosis (Hosokawa *et al*, 2010). Indeed, recent data show that Avastin in combination with the standard treatment did not improve overall patient survival compared to standard treatment alone (Gilbert *et al*, 2014); in addition, the group receiving Avastin experienced a significant impact in terms of angiogenic side effects. Avastin did improve progression-free survival to 10.6 months up from 6.2 months as reported by Genentech as part of the AVAglio phase III study. Prevention of GBM progression while not improving overall survival suggests that Avastin is more a means to contain GBM growth, rather than eliminate the tumor. As such, Avastin may be more useful for mitigating early-stage progression. An independent investigation showed that VEGF blockade reduced tumor size as expected, but unfortunately bolstered tumor invasiveness in human and mouse models (de Groot *et al*, 2010), possibly due to starvation-induced stimulation of tumor cell escape. Avastin is nevertheless still considered one of the best new treatments for GBM due to the relatively limited added toxicity compared to standard treatment of care.

In contrast to tumor agnostic antibodies like Bevacizumab, AMG595 specifically targets EGFRvIII and is currently being tested in phase I clinical trials. AMG595 is a non-cleavable linker immunoconjugate between a human monoclonal antibody directed against EGFRvIII and the cytotoxic agent mertansine (DM1). Once AMG595 engages EGFRvIII, receptor-mediated internalization occurs, thus targeting cytotoxic DM1 to the tumor cells expressing EGFRvIII. AMG595 comes with its own set of limitations. As described previously, *EGFR* is mutated in roughly 40% of GBM cases. Of these, 65% have EGFRvIII mutations, which thus leaves only a limited percentage of the total GBM cases that can potentially benefit from AMG595 as a possible treatment option and only in those cases that can be easily identified through immunohistological staining of tumor biopsies or microvesicle detection.

While therapeutic antibodies carry great potential due to the inherent specificity of binding and the multitude of surface proteins, there are specific issues in the case of GBM (and other brain tumors). In fact, any drug administered systemically would require transport across the blood–brain barrier, which normally impedes access to the vast majority of drugs. There are, however, various endothelial uptake mechanisms, which may be exploited to make antibody delivery to brain tumors possible. The transferrin receptor mediates the transfer of ligands via iron-mediated endocytosis (Qian *et al*, 2002). Antibodies might be adapted to use this system for brain delivery by enhancing their affinity for the transferrin receptor and thus increase passage across the BBB.

### Innate immunotherapy

As an alternative approach, some groups are attempting to reengineer the patient innate immune system in order to combat their own GBM tumor. DCVax-L by Northwest Biotherapeutics is currently in phase III trial for newly diagnosed GBM cases. The phase I/II clinical trials showed that median life expectancy for DCVax-L-treated patients increased to nearly 3 years. DCVax-L uses patient-derived tumor and healthy dendritic cell tissues to educate the innate immune response to recognize GBM tissue for elimination and has been shown to be safe (Yu *et al*, 2004) (Prins *et al*, 2011). Differentiated dendritic cells are presented with the tumor biopsy and then reintroduced in the patient, thereby promoting T-cell aggregation and elimination of tumor cells. Prepared DCVax-L is administered intradermally three times with 2 week intervals between each administration. This is a clear example of personalized medicine as it requires immunization against a person's own tumor. DCVax-L immunization also requires tissue samples to be shipped to the DCVax-L laboratory for vaccine manufacturing.

A recent press release from Agenus on their Prophage G100 vaccine details the positive outcomes of a phase II trial (http://www.agenusbio.com). As with DCVax-L, clinicians use tumor biopsies to develop a personalized vaccine which induces the patient's T-cell population to eliminate the tumor. Heat-shock protein glycoprotein 96 and bound tumorigenic peptides partners are extracted from a patient's tumor and reintroduced intradermally to activate innate antigen-presenting cells, which expands the T-cell population. This vaccine is used in conjunction with the standard treatment of care. The released results of the phase II study indicate that the median survival rate had increased to 23.3 months from the 14.6 months with standard treatment alone. The positive outcome of the multi-institutional phase II study has clear promise as a combination therapy, but Prophage G100 has not entered a randomized phase III trial as of yet.

### Oncolytic viruses

Oncolytic viruses have potential use as a treatment for GBM. These viruses are replication incompetent except in specific cell populations such as tumors. Once the selected viruses find their host cell through surface marker identification, the viruses undergo lytic expansion, thus destroying the cell population, and remain replicative incompetent once the cell population is eradicated. After the tumor cell population is eliminated, patients can be treated with anti-viral medication to remove excess virus. These viruses are readily genetically manipulated and are effective unless the patient has a pre-existing immunity to the viral type used. Selectivity of these viruses depends on the cell surface expression of targeted receptors. EGFRvIII, PDGFR, and IL-13R have all been used as selectivity receptors for GBM in oncolytic virus production.

Oncolytic viruses are under investigation for use in GBM, including Herpes Simplex 1 as it contains double-stranded DNA and is a common infectious human pathogen. HSV-1 M032 is being explored as it lacks the y134.5 neurovirulence loci which prevents virus latency and has appropriate bio-distribution after intracerebral injection in non-human primates with no adverse clinical signs (Roth *et al*, 2014). HSV-1 M032 is in phase I trial, which has not been opened for recruitment to date. GBM Adenovirus trials have also begun using DNX-2401. Formerly known as Delta-24 this adenovirus is selective for GBM due to the deregulation of retinoblastoma protein in several cancers like GBM. Delta-24 replication is dependent on functionally inactive retinoblastoma protein (Fueyo *et al*, 2000). The addition of an RGD-4C peptide gives the virus high affinity for integrins αvβ3 and αvβ5 and increases oncolytic activity against GBM compared to non-RGD-containing analogs (Fueyo *et al*, 2003). Although the mechanism remains unclear, the adenovirus may promote cell death through autophagic activity as noted through the appearance of autophagic vesicles. In mouse xenograft models, Delta-24-RGD-4C therapy decreased tumor size and increased mouse survival. Preliminary results indicate that Delta-24-RGD-4C single-dose injections resulted in either stable, partial, or complete regression in 52% of GBM cases (Pol *et al*, 2013).

### Small-molecule inhibitors

Targeted screening has led to potential GBM small-molecule inhibitors such as NSC-154829 which selectively upregulates caspases 3 and 7 in EGFRvIII-expressing GBM cells promoting apoptotic death (Trembath *et al*, 2007). NSC-154829 does not have any downstream pathway effect and does not elicit a response in secondary GBM cell lines which may suggest caspases 3 and 7 as markers of primary GBM (Trembath *et al*, 2007). There is limited public data on NSC-154829 to estimate the feasibility of this small-molecule inhibitor as a treatment.

The WNT pathway inhibitor SEN461 is another potential small-molecule therapeutic target for GBM. The WNT pathway is not traditionally considered a GBM-relevant one; however, SEN461 was found to inhibit GBM growth both *in vitro* and *in vivo* (De Robertis *et al*, 2013). The compound interferes with β-catenin phosphorylation, which is required for the anchorage-dependent growth of GBM, and although its efficacy has been shown both in patient-derived cells and in a *Xenopus* embryo model *in vivo*, no clinical progress has been reported to date.

Screening of a NIH diversity set of 1364 compounds identified Vacquinol-1 as an inducer of non-apoptotic cell death in glioma cells (Kitambi *et al*, 2014). Cell death was the result of micropinocytotic vacuole accumulation, which led to redistribution of the cytoplasm causing cell membrane rupture. The effect of Vacquinol-1 appears glioma cell specific. While the exact mechanism is unknown, shRNA knockdown of MMK4, a factor critical in micropinocytosis, rendered glioma cells resistant to Vacquinol-1. Of relevance, the compound crossed the blood–brain barrier in a murine xenograft model of GBM, where it significantly increased survival providing positive preclinical validation of the compound. This novel and potentially effective compound may in turn provide a unique therapeutic strategy given its mode of action.

**Figure 2:**
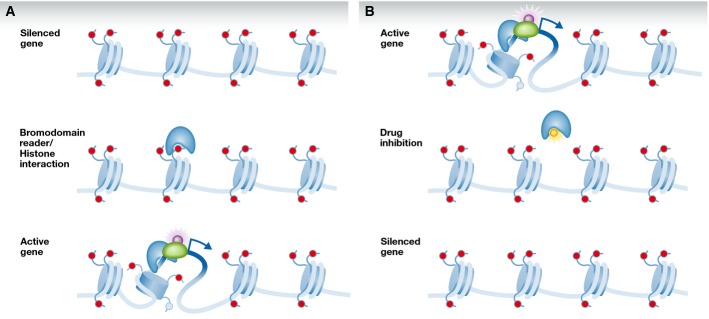
Drug modification of epigenetic regulation (A) Bromodomain readers recognize modified residues on histone tails which can lead to unraveling of the DNA/histone complex contingent on the bromodomain-containing complex composition. Unwound DNA is available for transcription complex interaction and transcription. (B) Unwound, transcriptionally active DNA reliant on bromodomain-containing complexes can be therapeutically targeted. Drugs blocking the bromodomain/histone tail modification interaction can prevent the helicase activity by bromodomain-containing complexes, thus stereohindering transcription regulators and silencing genes.

While the two small molecules described above are pathway inhibitors, small-molecule epigenetic modulators are also receiving considerable attention as a possible therapeutic option. Such compounds alter the epigenetic landscape and may impact many downstream pathways simultaneously. For instance, epigenetic drugs may affect tumor growth by regulating gene expression through the availability of heterochromatin. Bromodomain (BRD)-containing proteins are sensors that bind to acetylated lysines on histone residues and recruit protein complexes to alter gene expression by modulating heterochromatin (Sanchez & Zhou, 2009). The inhibition of epigenetic readers can prohibit complex formation and subsequent transcription (Fig 2). JQ1, an inhibitor of the Bromodomains and extra terminal (BET) domain family of proteins, has been shown to reduce GBM size in mice, which might be of clinical relevance even though JQ1 is unlikely to be useful in the clinic due to its short half-life and low CNS delivery (Cheng *et al*, 2013b). JQ1, however, is but one of many BET inhibitors that are currently under investigation. For instance, IBET-151 or IBET-762 is currently being investigated in GBM as a possible alternative, although they are not brain penetrant (Dawson *et al*, 2011). For instance, Bromodomain 4 disruption using the small-molecule inhibitor IBET-151 led to GBM cell cycle arrest in cell line models (Pastori *et al*, 2014). Epigenetix Inc. appears to have brain-penetrant long-lasting BRD inhibitors useful for GBM, though no clinical trials have been initiated yet (epigenetix.com, personal communication). No epigenetic small-molecule inhibitor is currently under clinical trial for GBM; it is therefore impossible to assess this approach in terms of treatment outcomes. It is, however, clear that epigenetic regulation plays an important role in tumorigenesis, and thus, this approach is one of the most exciting potential developments in GBM therapy development, and we eagerly anticipate future clinical trials in this space.

## Conclusion

Current GBM treatments have not improved overall patient survival rates to the levels achieved for other brain tumors. From the basic science standpoint, there is a critical need to understand how GBM arises or is evolved from earlier gliomas. Targeted therapies may prove to have limited efficacy as GBM can arise from a variety of mutations. Early diagnosis may be the key to improving patient survival rates through the prevention of tumor growth, and therefore, the identification of early biomarkers is critical. Non-invasive blood monitoring of tumor microvesicles may provide quick, accurate, and early detection of GBM. Early subtyping of GBM tumors could occur before patients undergo tumor resection to identify treatment regimens that may reduce tumor volume pre-resection. The combination of treatment approaches detailed here may prove an effective regime for the treatment of GBM tumors.

We suggest, for example, that early detection of an EGFRvIII-presenting GBM, diagnosed through blood microvesicle screening, could perhaps one day allow a therapeutic regimen that would extend patient survival well beyond current levels. Such a patient could also be given epigenetic drugs to arrest differentiating GBM tumor cells and prevent tumor growth and development in a neoadjuvant setting. Indeed, should the tumor in such a patient be located distally from the SVZ and the patient undergoes dye-assisted resection, followed by a personalized vaccine, then prognosis is likely to be even better. Current TMZ and radiotherapy treatment options to kill the remaining tumor cells will continue to be used to prevent any possibility of recurrence. Considering all the possible new treatments that are under investigation, we posit that we are on the verge of a watershed moment in GBM management.

## Abbreviations

2-hydroxyglutarate: Isocitrate is metabolized to alpha-ketoglutarate by IDH-1. Mutated IDH-1 (with a histidine substitution at arginine 132) can further metabolize alpha-ketoglutarate to 2-hydroxyglutarate. This onco-metabolite has been associated with a dysregulation in reactive oxygen species.
Aerobic Glycolysis: The conversion of glucose to lactic acid even in the presence of oxygen
Adenovirus: Non-enveloped virus that contains single-stranded double DNA capable of using host machinery to propagate
Biomarker: Cellular component capable of predicting the biological state of a cell population
Bromodomain: Alpha helical protein domain that recognizes acetylation marks on histone tails
Epigenetics: The study of transcriptional regulation not caused by DNA sequence. Epigenetic changes can lead to transient or sustained alterations to gene expression based on the type of modification such as histone acetylation or DNA methylation.
Pericyte: Vasculature-wrapping cells critical in maintaining the blood–brain barrier through interactions with endothelial cells, neurons, and astrocytes. G-pericytes are those derived from glioblastoma multiforme.
Gamma Knife: Stereotactic high-dose radiation targeting the brain region of interest
Immunoconjugate: Antibody conjoined to a second molecule, usually cytotoxic, in order to direct the cytotoxin to the target of interest
Microvesicle: Plasma membrane-derived membrane-enclosed bodies that may contain miRNA, mRNA, or protein, and are capable of intercellular communication.
Perfusion Weight Imaging: Contrast dye visualization of cerebral blood flow in vessels using magnetic resonance imaging
